# Rural-Urban Parity and Socioeconomic Gaps in Traumatic Brain Injury Outcomes

**DOI:** 10.5811/westjem.62017

**Published:** 2026-09-05

**Authors:** Minahil Cheema, Aamna Cheema, Anastasia Ternovskaia, Raneem Hamada Megahed, Patrick McGinnis, Japneet Sarai, Taylor Kowansky, Shriya Jaddu, Emily Esposito, Jessica V Downing, Quincy K Tran

**Affiliations:** * University of Maryland School of Medicine, Baltimore, Maryland; † University of Maryland School of Medicine, Research Associate Program in Emergency Medicine and Critical Care, Department of Emergency Medicine, Baltimore, Maryland; ‡ George Washington University, School of Medicine and Health Sciences, Washington, DC; § University of Maryland School of Medicine, Department of Emergency Medicine, Baltimore, Maryland; || University of Maryland School of Medicine, Program in Trauma, R Adams Cowley Shock Trauma Center, Baltimore, Maryland

## Abstract

**Background:**

Traumatic brain injury (TBI) is a leading cause of death and disability in the United States. Rural residence and low community income are independently associated with poorer health outcomes, mediated by variations in injury mechanism and healthcare access. We evaluated the impact of geographic location on injury patterns and acute-care metrics in adults with TBI treated at Maryland’s statewide Level I trauma center.

**Methods:**

Adults (≥18 years of age) with radiographically confirmed TBI admitted between 2017 and 2021 were identified retrospectively from the institutional trauma registry. Rural residence was defined using Rural-Urban Commuting Area codes. This single-center study included only patients surviving to hospital admission. Primary outcomes included hospital length of stay (LOS) and discharge to hospice or death. Secondary outcomes included intensive care unit (ICU) and ventilator days and discharge disposition.

**Results:**

Of 2,812 patients diagnosed with TBI during the study period, 267 (9.5%) resided in rural areas. Compared with nonrural patients, rural patients were older (median age 67 vs 60 years, *P* < .001) and lived in lower income communities. However, after adjustment for demographic and clinical covariates, rural residence was not associated with higher hospital LOS quartile category (adjusted odds ratio [aOR] 1.18; 95% CI, 0.91–1.53; *P* = .20), discharge to hospice or death (aOR 1.67; 95% CI, 0.95–2.94), or discharge to a nonhome setting (aOR 0.93; 95% CI, 0.66–1.31). Groups showed no significant differences in critical care use, including ICU and ventilator durations. Worse outcomes were associated primarily with injury severity, including lower Glasgow Coma Scale scores and higher Injury Severity Scores.

**Conclusion:**

In a well-established regional trauma network, we found that among patients admitted with traumatic brain injury, residing in a rural area was not independently associated with worse in-hospital outcomes compared to patients who resided in nonrural (urban and suburban) communities. These findings suggest that a coordinated statewide trauma triage and access to tertiary neurotrauma care may help other systems similarly reduce rural disparities after severe head injury.

## INTRODUCTION

Traumatic brain injury (TBI) accounts for millions of US emergency department visits annually and contributed to approximately 69,473 deaths in 2021 alone.^1^ Growing evidence suggests that a patient’s geographical location and socioeconomic status (SES) can substantially influence TBI outcomes.^2^ Rural residents sustain proportionally more high-velocity mechanism injuries (eg, motor vehicle collisions) and often experience longer prehospital times and limited geographical access to Level I trauma centers.^3^,^4^ Urban patients tend to be older, experience more fall-related injuries, and live in closer proximity to specialty care.^5^ A recent systematic review of approximately 2.5 million patients found that patients who resided in rural areas had 28% higher odds of severe TBI (defined by Glasgow Coma Scale [GCS] ≤ 8) and were less likely to achieve optimal functional recovery compared to their urban counterparts.^5^ Some studies also report higher TBI mortality in rural areas, potentially related to longer transport distances and fewer healthcare resources.^6^,^7^

Despite these disparities, the literature remains mixed. Several studies suggest that once rural patients reach tertiary centers within a well-organized trauma network, their outcomes may be similar to those of urban patients.^8^,^9^ This raises the possibility that coordinated trauma system organization, such as standardized statewide triage protocols, rapid interfacility transfer pathways, and helicopter emergency medical services (HEMS) may mitigate rural disadvantages after severe injury.

To address these gaps, our study examined the association between rural residence, adult TBI injury patterns, hospital course, and discharge outcomes among adults with TBI treated at Maryland’s statewide referral Level I trauma center. Because outcomes at a tertiary center are influenced by referral bias (thus capturing patients who survive initial injury and are triaged to high-level care), we sought to evaluate whether a regionalized model of coordinated field triage and HEMS is associated with rural-urban parity in hospital metrics, such as hospital length of stay (LOS), intensive care unit (ICU) use, ventilator use, and discharge disposition.

## METHODS

### Study Design and Setting

We performed a retrospective observational study of adult patients (≥18 years) treated for TBI at a statewide referral Level I trauma center. This study adhered to the Strengthening the Reporting of Observational Studies in Epidemiology (STROBE) guidelines. The facility is an integrated, free-standing trauma center that serves as the state’s Primary Adult Resource Center.^10^ Field triage is coordinated through the Maryland Institute for EMS Systems, which uses unified triage criteria to transport severely injured patients. Direct process measures, such as individual helicopter flight times and scene-triage intervals, were not available in the registry and are addressed as a study limitation.

**Population Health Research Capsule**

What do we already know about this issue?

*Rural residents suffer from disproportionately higher traumatic brain injury (TBI) mortality rates due to prolonged transport times and limited local resources*.

What was the research question?

*Does an integrated regional trauma network eliminate rural-urban disparities in acute TBI outcomes?*

What was the major finding of the study?

*Rurality was not significantly associated with a higher length-of-stay quartile (adjusted odds ratio 1.18; 95% CI, 0.91–1.53; P = .20)*.

How does this improve population health?

*Centralized neurotrauma hubs paired with coordinated regional field triage and aeromedical systems can potentially overcome rural-urban disparities*.

The study was approved by the institutional review board with a waiver of informed consent owing to its retrospective design.

### Maryland’s Social Geography

Maryland is a small, topographically diverse state anchored by two major urban hubs (Baltimore and the Washington, DC, metropolitan area), and extensive rural or medically underserved pockets—the Eastern Shore across the Chesapeake Bay, the Appalachian panhandle in the far west, and numerous lower density counties between the urban corridors (Figure 1). These rural counties typically have lower median household incomes and higher rates of Health Professional Shortage Areas (HPSA) than the state average. The Shock Trauma Center catchment area includes both densely populated urban ZIP codes and dispersed rural communities with limited local trauma resources.

### Patient Identification

We queried the institution’s prospectively maintained trauma registry, which captures more than 95% of admissions. We included patients admitted between January 1, 2017, and 31 December 31, 2021, with an isolated traumatic brain injury—defined as a head Abbreviated Injury Scale (AIS) score ≥ 3 and an AIS score. ≤ 2 in every other body region. Intracranial pathology was radiographically confirmed and included subdural hematoma, epidural hematoma, and traumatic subarachnoid hemorrhage. We excluded records lacking a residential ZIP code (9.1%) because rurality depends on that field. We analyzed these excluded records and found no significant demographic differences compared to the included cohort, suggesting that missingness was at random rather than tied to factors such as homelessness; the final analytic cohort consisted of 2,812 patients (Figure 2).

### Variables and Data Sources

Data abstraction followed Worster and Bledsoe’s guidelines. To ensure data quality, two trained abstractors independently reviewed a 10% random sample of records, achieving an interrater reliability (κ = .92); any discrepancies were resolved by a third senior adjudicator. Direct process measures, such as individual helicopter flight times and scene-triage intervals, were not available in the registry and are addressed as a study limitation.^11^

### Rurality

We mapped home ZIP codes to counties and census tracts and classified “rural” according to the definition established by the Federal. Office of Rural Health Policy of the US Health Resources and Services Administration (HRSA). Under this definition, a location is rural if it meets any of the following criteria: (1) the county is nonmetropolitan; (2) the census tract lies in a metropolitan county but carries a Rural-Urban Commuting Area (RUCA) code 4–10; or (3) the tract is at least 400 square miles, a population density of 35 or fewer persons per square mile and has a RUCA code 2–3. All other ZIP codes were classified as nonrural based on the Maryland Rural-Urban Classification. We excluded patients with unknown ZIP codes in sensitivity analyses.

### Medically Underserved Areas

The HRSA database was queried by patient home address to identify HPSA and Medically Underserved Areas (MUA).^12^ ZIP codes falling within a Maryland Health Enterprise Zone (MHEZ), which are census tracts designated as medically underserved by the Maryland Department of Health, were flagged to explore overlap between rurality, SES, and healthcare access.

### Outcomes

Hospital LOS, which is a patient-centered measure of morbidity and resource use that allows comparison across trauma populations, was designated a priori as the primary outcome and informed our sample-size calculation. Secondary outcomes examined both injury patterns and clinical courses. We compared (1) the distribution of intracranial injury types (subdural hematoma [SDH], subarachnoid hematoma [SAH], and epidural hematoma [EDH]; (2) initial injury severity by admission GCS score; (3) use of neurosurgical interventions (eg, craniotomy, craniectomy, external-ventricular–drain placement); (4) intensive-care metrics (ICU LOS and ventilator days); and (5) hospital discharge disposition (eg, home, short-term acute posthospital care, long-term subacute rehabilitation, death/hospice). Each outcome was analyzed uniformly across both independent variables: rurality; and community-income bracket.

### Sample Size Calculation

We based our sample size calculation for the primary outcome of hospital LOS according to Yue et al.^13^ Their study reported a median hospital LOS for patients with TBI as three days (interquartile range [IQR] 2–8) or a mean of four days (SD 2). Assuming a hospital LOS difference of one day between rural versus urban population, we calculated a sample size of 63 patients per group, or 126 total patients, to achieve a power of 80%, with statistical significance set at α = .05. While this analytical target was met for the primary outcome, the substantial baseline imbalance between the nonrural (*n* = 2,545) and rural (*n* = 267) cohorts inherently restricts the statistical power available for secondary clinical endpoints.

### Statistical Analysis

We conducted all statistical analyses using Minitab Statistical Software (Minitab, LLC, State College, PA). We used descriptive statistics to summarize patient characteristics by rurality, MHEZ, MUA, and HPSA status. Continuous variables were summarized as medians with IQR, and categorical variables as frequencies and percentages. Group comparisons were conducted using two-sample *t* tests or Mann-Whitney *U* tests for continuous variables and chi-square or Fisher exact tests for categorical variables, as appropriate.

Binary logistic regression models were then used to assess associations between independent variables and two binary outcomes: (1) discharge to death or hospice versus all other dispositions; and (2) discharge to a location other than home versus discharge home. An ordinal logistic regression was used to evaluate the primary outcome of LOS, which was categorized into quartiles: 0 = first quartile (≤ 1.07 days); 1 = second quartile (1.08–3.58 days); 2 = third quartile (3.59–8.84 days); and 3 = fourth quartile (> 8.84 days). All regression models were adjusted for demographic, clinical, and geographic covariates, including age, comorbidities, Injury Severity Score (ISS), rurality indicators, and ZIP code-level socioeconomic characteristics (Table 3). Variable inclusion was guided by clinical relevance and bivariate associations. Model outputs are provided in supplementary Appendices A–C.

## RESULTS

### Patient Characteristics

Between 2017 and 2021, 2,812 adults with isolated TBI were admitted, originating from diverse geographic areas of Maryland (Figure 1). Two-thirds were male, and the overall median age was 60 years. Only 267 patients (9.5 %) resided in rural ZIP codes. Rural patients were older (median 67 versus 60 years of age) and came from markedly lower-income communities (median annual ZIP code income: $71,600 vs $92,500) compared with nonrural peers; they were also more likely to be White (80 % vs 60 %) (Table 1).

### Injury Patterns

Subdural hematoma was the most common traumatic brain lesion, present in approximately 67% of all patients. The distribution of SDH and the need for neurosurgical procedures (craniotomy, craniectomy, external ventricular drain) did not significantly differ between rural and nonrural patients (all *P* > .20, Table 2).

### Hospital Course and Outcomes - Rural versus Nonrural

Unadjusted outcomes were largely equivalent (Table 2). Median hospital LOS was 3.6 days (1.1–9.0) for nonrural patients, and 3.3 days (0.84–7.8) for rural patients (*P* = .19). Use of ICU was similar, although rural patients had slightly longer total ICU time (median 0 [0–2.7] days versus 0 [0–3.3] days; *P* = .58) and comparable days spent on mechanical ventilation (0 days [0–2] for both groups, and *P* = .93). In-hospital mortality or hospice discharge trended higher among rural patients (15% versus 11%, *P* = .07) but did not reach significance (Table 2).

### Multivariable Analyses

After adjusting for demographics, comorbidities, and injury severity, rural residence was not associated with longer LOS (adjusted odds ratio [aOR] = 1.18; 95% CI, 0.91–1.53; *P* = .20), discharge to a non-home setting (aOR = 0.93; 95% CI, 0.66–1.31; *P* = .66), or death/hospice (aOR = 1.67; 95% CI, 0.95–2.94; *P* = .04) (Appendices A–C). In the multivariable ordinal logistic regression, diabetes (aOR = 1.33; 95% CI, 1.09–1.64; *P* = .01) and cerebral edema (aOR = 1.33; 95% CI, 1.01–1.76; *P* = .04), were associated with increased odds of belonging to a higher LOS quartile (Appendix A).

For discharge to death or hospice, significant associated factors included hypertension (aOR = 1.95; 95% CI, 1.30–2.90; *P* < .001); cardiac disease (aOR = 3.14; 95% CI, 2.11–4.70; *P* < .001)’ SAH (aOR, 1.99; 95% CI, 1.39–2.85; *P* < .001); and cerebral edema (aOR = 1.74; 95% CI, 1.07–2.82; *P* = .03). Higher Injury Severity Score (ISS) (aOR = 1.07 per point; 95% CI, 1.04–1.10; *P* < .001) and lower admission GCS (aOR = 0.80; 95% CI, 0.77–0.84; *P* < .001) were also strongly associated with increased odds of death/hospice disposition (Appendix B).

For nonhome discharge, significant covariates included hypertension (aOR = 2.12; 95% CI, 1.72–2.63; *P* < .001); cardiac disease (aOR = 1.82; 95% CI, 1.40–2.36; *P* < .001); neurological disease (aOR = 1.52; 95% CI, 1.23–1.89; *P* < .001); bacteremia (aOR = 6.16; 95% CI, 2.50–15.19; *P* < .001); SDH (aOR = 1.27; 95% CI, 1.02–1.59; *P* = .03); SAH (aOR = 1.67; 95% CI, 1.37–2.04; *P* < .001); and cerebral edema (aOR = 1.58; 95% CI, 1.07–2.34; *P* = .02). Epidural hematoma was associated with lower odds of nonhome discharge (aOR = 0.56; 95% CI, 0.40–0.80; *P* < .001). Higher ISS (aOR = 1.07 per point; 95% CI, 1.05–1.08; *P* < .001) and lower admission GCS (aOR = 0.77; 95% CI, 0.74–0.80; *P* < .001) also remained strongly associated with nonhome discharge (Appendix C).

Supplemental analyses examining HPSA, MUA, and MHEZ designations demonstrated substantial demographic and socioeconomic differences across groups but generally similar short-term hospital outcomes (Supplementary Appendices D–F). Patients residing in MUAs demonstrated greater acute-care use, including longer trauma resuscitation unit times (466 versus 432 minutes, *P* < .001); increased ICU use (median, 0 [0–4.4] versus 0 [0–2.5] ICU days; *P* = .03); and higher craniotomy rates (5% versus 3%; *P* = .02), despite similar LOS and discharge disposition outcomes (Appendix D). Subgroup analyses of HPSA and MHEZ similarly showed minimal differences in major in-hospital outcomes (Appendix E, F).

Overall, within Maryland’s coordinated trauma network, rural patients who survived to tertiary neurotrauma admission experienced care processes and short-term outcomes comparable to urban peers, despite older age and lower community income levels. Panels detailing MHEZ and MUA subanalyses are provided in the supplementary Appendices D–F.

## DISCUSSION

### Principal Findings

In a five-year, statewide cohort of 2,812 adults with isolated TBI, rural residence (9.5% of cases) was not associated with longer hospital or ICU stays, greater ventilator use, or increased odds of death or hospital discharge after adjustment. Rural patients were older and came from lower-income ZIP codes, yet their acute outcomes mirrored urban peers.

### Rural-Urban Parity: Potential Impact of an Inclusive Trauma System

In contrast to national trends, there were no significant rural-urban disparities in acute TBI outcomes. This finding is noteworthy given that, across the US, rural residents face substantially higher TBI-related mortality rates than their urban counterparts (27.5 versus 17.4 per 100,000 in 2017), with an estimated 23% higher fatality risk.^14^ Prior studies attribute this rural disadvantage to longer prehospital transport times, limited access to trauma centers, and delays in definitive care^6^,^15^; each five-mile increase in distance from a trauma center increases odds of death by approximately 8%.^16^

We also examined whether differences in the type or severity of intracranial injury might account for rural-urban variation in presentation.^17^ The distribution of SDH, SAH, and EPH, was nearly identical between rural and nonrural patients (all *P* > .05), and both median ISS and admission/pretransfer GCS were comparable (Table 2). These findings suggest that neurologic status at presentation is unlikely to be driven by systematically different injury patterns and may instead reflect other prehospital factors (eg, time from injury to intubation, hypotension, hypoxia) or baseline comorbidities.

Several features of Maryland’s trauma system may have contributed to the absence of observed rural-urban differences in admitted patients. First, the statewide helicopter-based network operated by the Maryland Institute for EMS Systems^18^ provides rapid scene or interfacility transport from any county to the Shock Trauma Center (Appendix G), or another designated trauma hospital, typically within the “golden hour.”^19^ This publicly funded HEMS program integrates first response through tertiary care, ensuring equitable access across the state. Second, direct admission to a high-volume Level I trauma center with 24/7 neurosurgical coverage standardizes definitive care.^20^ Similar patterns have been reported elsewhere: when rural patients are air-lifted directly to Level I facilities, survival approximates (or exceeds) that of urban ground-transport patients.^21^

In our cohort, these system-level features appear to have neutralized geography-driven disadvantages, echoing smaller single-center reports that once rural patients reach a high-level facility, outcomes converge with urban norms, emphasizing the value of rapid aeromedical transport and centralized expertise.^22^ Maryland’s coordinated model—statewide HEMS, mandated field triage, and a single adult Level I hub (illustrated in Figure 3)—transports severely injured patients within 30 minutes and may serve as a model for other states seeking to eliminate rural-urban disparities in trauma outcomes.

In our cohort, where acute care protocols were uniform, these disparities likely stem from upstream factors such as community-level injury mechanisms, comorbidities, preinjury health, and downstream determinants such as access to rehabilitation and postacute navigation. Trauma systems alone cannot erase such gradients; community-level prevention, insurance expansion, and postacute navigation programs are needed.

### Implications for Rural Health Equity

1. System design matters. A coordinated statewide trauma network may substantially reduce in-hospital rural disparities after severe injury. States with large rural catchments may benefit from coordinated trauma-system elements, including publicly funded HEMS, standardized statewide EMS triage protocols, and designated neurotrauma hubs (Figure 3).
2. Equity requires dual focus. However, ensuring geographic access alone is unlikely to eliminate broader disparities in recovery. Community-level prevention, rehabilitation access, insurance coverage, and postacute care navigation likely remain important determinants of long-term rural TBI outcomes.

## LIMITATIONS

This retrospective, single-center study may not generalize to regions without Maryland’s dense, publicly funded helicopter-based trauma network. Because the state is geographically compact and uses a unified state-operated aeromedical fleet, our findings likely represent a best-case rural scenario. Larger states, or those relying on fragmented, privatized EMS models, may face different logistical barriers. Analyses ended at hospital discharge; functional recovery, rehabilitation access, and long-term mortality were unavailable. Additionally, there are residual confounding variables—such as triage patterns, transport times, helicopter use, and transfer pathways inherent to retrospective studies—that may persist despite adjustment and influence outcomes.

Our study is constrained by a nearly 10-fold disparity in patient volume between cohorts (nonrural *n* = 2,545 vs rural *n* = 267). While the sample size was sufficient to demonstrate that statistical power was fully met for our primary metric (hospital LOS), this severe numerical imbalance leaves the study underpowered to detect subtle differences in low-incidence secondary endpoints. Specifically, these “rare outcomes” include low-frequency clinical complications and specialized surgical interventions, such as craniectomies (*n* = 0 in the rural cohort), cranioplasties (*n* = 1), or postinjury bacteremia (*n* = 5), where absolute event counts are too minimal to draw definitive comparison conclusions. Furthermore, our database omits prehospital deaths, which could still disproportionately disadvantage remote communities. Similar tertiary hospital-level parity has been reported only sporadically (eg, in Saskatoon, Canada, where authors noted that excluding prehospital deaths may partly erase a rural gap).^22^ Nevertheless, demonstrating parity among admitted patients provides a realistic benchmark for other regions aiming to close rural gaps through system design.

Additionally, our trauma registry output provided continuous critical care durations rather than categorical admission flags, preventing the calculation of the exact percentage of patients admitted to the ICU or requiring mechanical ventilation. Because our cohort included a high volume of mild-to-moderate TBIs (median admission GCS 14) that did not require critical care, the overall median for ICU and ventilator days was skewed to zero, limiting our ability to analyze critical care use as a binary outcome.

### Future Directions

Multicenter studies that integrate prehospital metrics, granular SES data, and long-term outcomes are needed to confirm whether the hospital-level parity observed here persists across diverse settings and throughout the continuum of recovery. Future work should replicate these findings in larger, more geographically diverse settings; quantify prehospital mortality to confirm complete rural equity; and test interventions (eg, targeted prevention campaigns in low-income communities and postacute navigation services) aimed at narrowing SES-related gaps.

## CONCLUSION

In this statewide cohort of adults with traumatic brain injury treated within a centralized Level I trauma system, rural residence was not independently associated with worse short-term in-hospital outcomes despite older age and lower community income among rural patients. These findings suggest that coordinated statewide trauma organization, including rapid aeromedical transport, standardized triage pathways, and centralized neurotrauma care, may help mitigate rural disparities once patients successfully access tertiary treatment. However, persistent differences in prehospital access, rehabilitation availability, and broader social determinants of health likely continue to influence long-term rural TBI outcomes beyond the acute hospitalization period.

## Supplementary Information



## Figures and Tables

**Figure 1:**
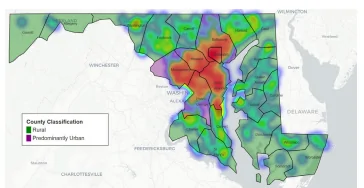
Patient density heatmap of the state of Maryland by rurality and home ZIP code in a study of traumatic brain injury outcomes at a regional Level I trauma center.

**Figure 2:**
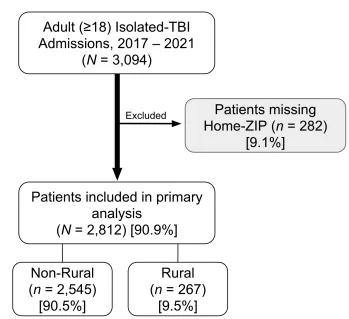
Flow diagram for patient selection in a study of traumatic brain injury outcomes at a regional Level I trauma center. *TBI*, traumatic brain injury.

**Figure 3:**
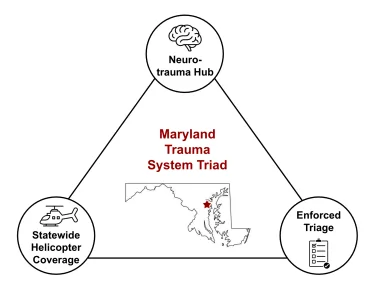
Maryland trauma system triad: neurotrauma hub; statewide helicopter coverage; and enforced field triage in a study of traumatic brain injury outcomes at a regional Level I trauma center. The three cornerstones of the Maryland’s integrated trauma network are depicted as a hub-and-spoke triangle. The red star marks the location of the R. Adams Cowley Shock Trauma Center in Baltimore, the state’s sole adult Level I trauma hub.

**Table 1:** Rural versus nonrural baseline characteristics in a study of traumatic brain injury outcomes at a regional Level I trauma center.

Variables	All Patients (*N* = 2,812)	Rurality				

		Nonrural (*n* = 2,545)	Rural (*n* = 267)	Diff	95% CI	*P*
Demographics						
Female (%)	900 (32)	808 (32)	92 (34)	−0.03	−0.09 to 0.03	.38
Male (%)	1,911 (68)	1,736 (68)	175 (66)	0.03	−0.03 to 0.09	.38
Age, median [IQR]	60.5 [40–77]	60 [40–77]	67 [47–81]	−5	−7 to −2	< .001
Median household income [$]	89,398 [61,572–112, 065]	92,527 [61,572–114, 409]	71,629 [59,135–87, 611]	15,036	11,421–19,229	< .001
Race / Ethnicity (%)						
White	1,731 (62)	1,518 (60)	213 (80)	−0.2	−0.25 to −0.15	< .001
Black	842 (30)	797 (31)	45 (17)	0.14	0.10–0.19	< .001
Asian	59 (2)	58 (2)	1 (0.3)	0.02	0.01–0.03	.04
Native Hawaiian / Pacific Isl.	4 (0.1)	4 (0.2)	0 (0)	0.002	0.0–0.003	> .99
American Indian / Alaskan Native	6 (0.2)	6 (0.2)	0 (0)	0.002	0.0004–0.004	> .99
Other / ≥2 races	133 (5)	126 (5)	7 (3)	0.02	0.002–0.04	.03
Comorbidities (%)						
Hypertension	1,298 (46)	1,180 (46)	118 (44)	0.02	−0.04 to 0.08	.50
Diabetes	469 (17)	429 (17)	40 (15)	0.02	−0.03 to 0.06	.42
Liver disease	125 (4)	109 (4)	16 (6)	−0.02	−0.05 to 0.01	.26
Cardiac disease	504 (18)	454 (18)	50 (19)	−0.01	−0.06 to 0.04	.72
Kidney disease	97 (3)	91 (4)	6 (2)	0.01	−0.006 to 0.03	.37
Neurological disorder	744 (26)	674 (26)	70 (26)	0.003	−0.05 to 0.06	.93
Alcohol / drug use	246 (9)	217 (9)	29 (11)	−0.02	−0.06 to 0.02	.24
Prior surgery	1,017 (36)	923 (36)	94 (35)	0.01	−0.05 to 0.07	.73
Top 5 Injury Mechanisms (%)						
Fall	1,777 (63)	1,615 (63)	162 (61)	0.03	−0.03 to 0.09	.37
ATV accident	398 (14)	354 (14)	44 (16)	−0.03	−0.07 to 0.02	.28
Assault	329 (12)	293 (12)	36 (13)	−0.02	−0.06 to 0.02	.37
Pedestrian accident	101 (4)	95 (4)	6 (2)	0.01	−0.004 to 0.03	.13
Inanimate mechanical force	63 (2)	54 (2)	9 (3)	−0.01	−0.03 to 0.01	.27

*ATV*, all-terrain vehicle; *Diff*, unadjusted absolute difference between the two comparison groups; *IQR*, interquartile range.

**Table 2:** Clinical course and outcomes comparing rural versus nonrural patients in a study of traumatic brain injury outcomes at a regional Level I trauma center.

	All Patients (*N* = 2,812)	Rurality				

		Nonrural (*n* = 2,545)	Rural (*n* = 267)	Diff	95% CI	*P*
Injury (%)						
Subdural hematoma	1,885 (67)	1,703 (67)	182 (68)	−0.01	−0.07 to 0.05	.67
Subarachnoid hemorrhage	1,251 (44)	1,134 (44)	117 (44)	0.007	−0.05 to 0.07	.82
Epidural hematoma	289 (10)	267 (10)	22 (8)	0.02	−0.01 to 0.06	.21
Edema	239 (8)	210 (8)	29 (11)	−0.03	−0.06 to 0.01	.19
Bacteremia	88 (3)	83 (3)	5 (2)	0.01	−0.004 to 0.03	.12
Clinical Variables [IQR]						
Length of stay (days)	3.6 [1.1–8.8]	3.6 [1.1–9.0]	3.3 [0.84–7.8]	0.21	−0.10 to 0.61	.19
Ventilator days	0 [0–2]	0 [0–2]	0 [0–2]	0	0–0	.93
TRU minutes	451 [281–765]	451 [283–771]	444 [255–700]	23.5	−16.7 to 64.4	.25
Total ICU Days	0 [0–3.3]	0 [0–3.3]	0 [0–2.7]	0	0–0	.58
Glasgow Coma Scale	14 [11–15]	14 [11–15]	14 [9–15]	0	0–0	.54
PTA GCS	13 [7–15]	13 [7–15]	13 [7–14]	0	0–1	.17
Injury Severity Score	17 [10–25]	17 [10–25]	16 [10–25]	0	0–0	.50
TRISS	0.95 [0.88–0.98]	0.95 [0.88–0.98]	0.95 [0.87–0.98]	0	−0.002 to 0.005	.68
White Blood Cell Count (×1000 counts/μL)	9.9 [7.5–13.4]	9.9 [7.4–13.4]	10.4 [7.8–13.2]	−0.2	−0.7 to 0.3	.47
Admission SBP	145 [130–169]	145 [130–170]	144 [129–166]	2	−2 to 5	.30
Admission DBP	84 [74–94]	84 [73–94]	84 [73–94]	0	−2 to 3	.65
Procedures						
Craniotomy	118 (4)	110 (4)	8 (3)	0.01	−0.009 to 0.04	.24
Craniectomy	17 (0.6)	17 (0.6)	0 (0)	0.007	0.004–0.01	.39
Burr hole	6 (0.2)	4 (0.2)	2 (0.7)	−0.006	−0.02 to 0.004	.10
Cranioplasty	12 (0.4)	11 (0.4)	1 (0.4)	0.001	−0.007 to 0.008	> .99
External ventricular drain	65 (2)	55 (2)	10 (4)	−0.02	−0.04 to 0.008	.19
CT Head	2,546 (90)	2,313 (91)	233 (87)	0.04	−0.005 to 0.08	.09
Radiograph of Head	13 (0.5)	12 (0.5)	1 (0.4)	0.001	−0.007 to 0.008	> .99
Discharge Disposition						
Death / Hospice	319 (11)	279 (11)	40 (15)	−0.04	−0.08 to 0.004	.07
Nonhome	1,420 (50)	1,290 (51)	130 (49)	0.02	−0.04 to 0.08	.54
Home	1,357 (48)	1,225 (48)	132 (49)	−0.01	−0.08 to 0.05	.68
Short-term acute posthospital care*	683 (24)	628 (25)	55 (20)	0.04	−0.01 to 0.09	.12
Long-term subacute care**	415 (15)	380 (15)	35 (13)	0.02	−0.02 to 0.06	.40

* Short-term acute post-hospital care includes those discharged to acute care, inpatient units, inpatient rehabilitation facilities.

** Long-term subacute care includes those discharged to psychiatric facilities, residential facilities, specialty referral centers, subacute rehabilitation facilities.

*CT*, computed tomography; *DBP*, diastolic blood pressure; *Diff*, unadjusted absolute difference between the two comparison groups; *ICU*, intensive care unit; *IQR*, interquartile range; *PTA GCS*, pretransfer or prehospital Glasgow Coma Scale; *TRISS*, Trauma and Injury Severity Score; *SBP*, systolic blood pressure; *TRU*, trauma resuscitation unit.

**Table 3:** List of all variables used in the multivariable logistic and multivariable ordinal regressions in a study of traumatic brain injury outcomes at a regional Level I trauma center.

Continuous variables	Categorical variables
Age	Sex
White blood cell count	Past medical history of hypertension
Mean arterial pressure	Past medical history of diabetes
Minute in trauma resuscitation unit	Past medical history of liver disease
Injury Severity Score	Past medical history of kidney disease
Trauma and Injury Severity Score	Past medical history of heart disease
GCS	Past medical history of neurological disease
ZIP code median income	Past medical history of drug/alcohol use Medical History of Past Surgeries Bacteremia Subdural Hematoma Subarachnoid Hemorrhage Epidural Hematoma Edema Race CT of Head Health Professional Shortage Area (HPSA) Medically Underserved Areas (MUA) Maryland Health Enterprise Zones (MHEZ) Rural Status

*CT*, computed tomography; *GCS*, Glasgow Coma Scale.
