# Supplementary material for: Rural-Urban Parity and Socioeconomic Gaps in Traumatic Brain Injury Outcomes
Source: West J Emerg Med. 2026 Sep 5;27(5):1271–9. doi: 10.5811/westjem.62017 (PMC13660562; doi:10.5811/westjem.62017)
Supplement: Supplementary file 1 [file wjem-27-1271-s001.docx]

**Appendix A**. Results from ordinal logistic regression assessing the association between patients’ demographic and clinical factors and the likelihood of being in a higher quartile of length of stay in days in a study of traumatic brain injury outcomes at a regional Level I trauma center. Length of stay was categorized into quartiles as follows:

0 = 1st quartile (LOS ≤ 1.07 days);

1 = 2nd quartile (1.07 < LOS. ≤ 3.58 days);

2 = 3rd quartile (3.58 < LOS ≤ 8.84 days);

3 = 4th quartile (LOS > 8.84 days).

| Variables | OR | 95% CI | *P* | Coefficient |
| --- | --- | --- | --- | --- |
| Age | 0.99 | 0.99-0.99 | <.001 | -0.01 |
| Rural | 1.18 | 0.91-1.53 | .20 | 0.17 |
| Past Medical History |  |  |  |  |
| Diabetes | 1.33 | 1.09-1.64 | .01 | 0.29 |
| Kidney Disease | 1.48 | 0.98-2.23 | .06 | 0.39 |
| Clinical Factors |  |  |  |  |
| Edema | 1.33 | 1.01-1.76 | .04 | 0.28 |
| Clinical Factors at Arrival: Continuous |  |  |  |  |
| ISS | 1.00 | 0.98-1.01 | .58 | -0.003 |
| TRISS | 0.93 | 0.51-1.73 | .84 | -0.06 |
| GCS | 1.00 | 0.98-1.03 | .79 | 0.004 |

Only the primary exposure, core injury-severity covariates, and statistically significant predictors are displayed. All variables listed in Table 3 were included in the multivariable model.

*LOS*, length of stay; *OR*, odds ratio; *CI*, confidence interval; *ISS*, Injury Severity Score; *TRISS*, Trauma and Injury Severity Score; *GCS*, Glasgow Coma Scale

**Appendix B**. Multivariable logistic regression assessing association between patients’ demographic and clinical factors and hospital disposition of death or hospice in a study of traumatic brain injury outcomes at a regional Level I trauma center.

| Variables* | OR | 95% CI | *P* | VIF |
| --- | --- | --- | --- | --- |
| Age | 0.99 | 0.99-1.00 | .22 | 1.17 |
| Female | 0.69 | 0.45-1.04 | .07 | 1.09 |
| MUA | 0.56 | 0.35-0.88 | .01 | 1.76 |
| Rural | 1.67 | 0.95-2.94 | .07 | 1.12 |
| Past Medical History |  |  |  |  |
| Hypertension | 1.95 | 1.30-2.90 | <.001 | 1.33 |
| Cardiac Disease | 3.14 | 2.11-4.70 | <.001 | 1.28 |
| Alcohol/Drug Use | 0.49 | 0.23-1.07 | .07 | 1.03 |
| Clinical Factors: Categorical |  |  |  |  |
| Subdural Hematoma | 1.54 | 0.95-2.50 | .08 | 1.15 |
| Subarachnoid Hemorrhage | 1.99 | 1.39-2.85 | <.001 | 1.13 |
| Edema | 1.74 | 1.07-2.82 | .03 | 1.08 |
| CT Scan of Head | 0.45 | 0.24-0.84 | .01 | 1.12 |
| Clinical Factors at Arrival: Continuous (each unit) |  |  |  |  |
| Minutes in TRU | 1.00 | 0.99-1.01 | .04 | 1.17 |
| ISS | 1.07 | 1.04-1.10 | <.001 | 1.23 |
| GCS | 0.80 | 0.77-0.84 | <.001 | 1.48 |

*Hosmer-Lemeshow chi square D(f) = 11.05; D(f)= 8; *P* = .19; AUROC = 0.88.

Only the primary exposure, core injury-severity covariates, and statistically significant predictors are displayed. All variables listed in Table 3 were included in the multivariable model.

*OR*, odds ratio; *CI*, confidence interval; *VIF*, variance inflation factor; *MUA*, Medically Underserved Area; *CT*, computed tomography; *TRU*, trauma resuscitation unit; *ISS*, Injury Severity Score; *GCS*, Glasgow Coma Scale; *AUROC*, area under the receiving operating characteristic curve

**Appendix C**. Multivariable logistic regression assessing association between patients’ demographic and clinical factors and hospital disposition of anywhere not home in a study of traumatic brain injury outcomes at a regional Level I trauma center.

| Variables* | OR | 95% CI | *P* | VIF |
| --- | --- | --- | --- | --- |
| Age | 0.99 | 0.99-1.00 | .23 | 1.15 |
| MUA | 0.69 | 0.53-0.89 | .01 | 1.80 |
| Rural | 0.93 | 0.66-1.31 | .66 | 1.08 |
| Past Medical History |  |  |  |  |
| Hypertension | 2.12 | 1.72-2.63 | <.001 | 1.26 |
| Cardiac Disease | 1.82 | 1.40-2.36 | <.001 | 1.16 |
| Neurological Disease | 1.52 | 1.23-1.89 | <.001 | 1.04 |
| Past Surgeries | 1.22 | 0.99-1.51 | .05 | 1.15 |
| Clinical Factors: Categorical |  |  |  |  |
| Bacteremia | 6.16 | 2.50-15.19 | <.001 | 1.01 |
| Subdural Hematoma | 1.27 | 1.02-1.59 | .03 | 1.17 |
| Subarachnoid Hemorrhage | 1.67 | 1.37-2.04 | <.001 | 1.09 |
| Edema | 1.58 | 1.07-2.34 | .02 | 1.03 |
| Epidural Hematoma | 0.56 | 0.40-0.80 | <.001 | 1.11 |
| CT Scan of Head | 1.43 | 0.99-2.07 | .06 | 1.08 |
| Clinical Factors at Arrival: Continuous (each unit) |  |  |  |  |
| ISS | 1.07 | 1.05-1.08 | <.001 | 1.17 |
| GCS total | 0.77 | 0.74-0.80 | <.001 | 1.12 |

*Hosmer–Lemeshow test chi-square 17.31, D(f) = 8; *P* = .03; AUROC = 0.81.

Only the primary exposure, core injury-severity covariates, and statistically significant predictors are displayed. All variables listed in Table 3 were included in the multivariable model.

*OR*, odds ratio; *CI*, confidence interval; *VIF*, variance inflation factor; *CT*, computed tomography; *ISS*, Injury Severity Score; *GCS*, Glasgow Coma Scale; *AUROC*, area under the receiving operating characteristic curve

**Appendix D1**. Medically underserved area baseline characteristics in a study of traumatic brain injury outcomes at a regional Level I trauma center.

| Variables | All  Patients  (N = 2,812) | MUA | | | | |
| --- | --- | --- | --- | --- | --- | --- |
|  |  | Non-MUA  (n = 1,618) | MUA  (n = 1,194) | Diff | 95% CI | *P* |
| Demographics | | | | | | |
| Female (%) | 900 (32) | 562 (35) | 338 (28) | 0.06 | 0.03-0.10 | <.001 |
| Male (%) | 1,911 (68) | 1,056 (65) | 855 (72) | -0.06 | -0.10 to -0.03 | <.001 |
| Age, median [IQR] | 60.5 [40-77] | 65 [42-79] | 57 [38-73] | 5 | 3-6 | <.001 |
| Median household income [$] | 89,398 [61,572-112,065] | 100,853 [80,692-119,154] | 59,098 [49,171-91,563] | 35,567 | 3,148-37,825 | <.001 |
| Rural (%) | 267 (9) | 88 (5) | 179 (15) | -0.1 | -0.12 to -0.07 | <.001 |
| Race / Ethnicity (%) | | | | | | |
| White | 1,731 (62) | 1,116 (69) | 615 (52) | 0.17 | 0.14-0.21 | <.001 |
| Black | 842 (30) | 350 (22) | 492 (41) | -0.19 | -0.23 to -0.16 | <.001 |
| Asian | 59 (2) | 42 (3) | 17 (1) | 0.01 | 0.001-0.02 | .03 |
| Native Hawaiian / Pacific Isl. | 4 (0.1) | 4 (0.2) | 0 (0) | 0.002 | 0.0-0.004 | .14 |
| American Indian / Alaskan Native | 6 (0.2) | 4 (0.2) | 2 (0.2) | 0.001 | -0.003 to 0.004 | >.99 |
| Other / 2+ Races | 133 (5) | 82 (5) | 51 (4) | 0.008 | -0.008 to 0.02 | .32 |
| Comorbidities (%) | | | | | | |
| Hypertension | 1,298 (46) | 761 (47) | 537 (45) | 0.02 | -0.02 to 0.06 | .28 |
| Diabetes | 469 (17) | 270 (17) | 199 (17) | 0.0002 | -0.03 to 0.03 | .98 |
| Liver disease | 125 (4) | 70 (4) | 55 (5) | -0.003 | -0.02 to 0.01 | .72 |
| Cardiac disease | 504 (18) | 296 (18) | 208 (17) | 0.009 | -0.02 to 0.04 | .55 |
| Kidney disease | 97 (3) | 57 (4) | 40 (3) | 0.002 | -0.01 to 0.02 | .80 |
| Neurological disorder | 744 (26) | 416 (26) | 328 (27) | -0.02 | -0.05 to 0.02 | .29 |
| Alcohol / drug use | 246 (9) | 147 (9) | 99 (8) | 0.008 | -0.01 to 0.03 | .46 |
| Prior surgery | 1,017 (36) | 578 (36) | 439 (37) | -0.01 | -0.05 to 0.03 | .57 |
| Top 5 Injury Mechanisms (%) | | | | | | |
| Fall | 1,777 (63) | 1,032 (64) | 745 (62) | 0.01 | -0.02 to 0.05 | .45 |
| ATV accident | 398 (14) | 242 (15) | 156 (13) | 0.02 | -0.007 to 0.04 | .15 |
| Assault | 329 (12) | 172 (11) | 157 (13) | -0.03 | -0.05 to -0.001 | .04 |
| Pedestrian accident | 101 (4) | 56 (3) | 45 (4) | -0.003 | -0.02 to 0.01 | .67 |
| Inanimate mechanical force | 63 (2) | 34 (2) | 29 (2) | -0.003 | -0.01 to 0.008 | .57 |

*MUA*, Medically Underserved Area; *Diff*, unadjusted absolute difference between the two comparison groups*; CI*, confidence interval; *IQR*, interquartile range; *ATV*, all-terrain vehicle

**Appendix D2**. Medically underserved area clinical course and outcomes in a study of traumatic brain injury outcomes at a regional Level I trauma center.

|  | All  Patients  (N = 2,812) | MUA | | | | |
| --- | --- | --- | --- | --- | --- | --- |
|  |  | Non-HPSA  (n = 1,618) | HPSA  (n = 1,194) | Diff | 95% CI | *P* |
| Injury (%) | | | | | | |
| Subdural hematoma | 1,885 (67) | 1,086 (67) | 799 (67) | 0.002 | -0.03 to 0.04 | .91 |
| Subarachnoid Hemorrhage | 1,251 (44) | 685 (42) | 566 (47) | -0.05 | -0.09 to -0.01 | <.001 |
| Epidural Hematoma | 289 (10) | 160 (10) | 129 (11) | -0.009 | -0.03 to 0.01 | .43 |
| Edema | 239 (8) | 134 (8) | 105 (9) | -0.005 | -0.03 to 0.02 | .63 |
| Bacteremia | 88 (3) | 42 (3) | 46 (4) | -0.01 | -0.02 to 0.001 | .06 |
| Clinical Variables | | | | | | |
| Length of Stay (days) | 3.6 [1.1-8.8] | 3.6 [1.1-8.7] | 3.5 [0.99-8.9] | 0.04 | -0.16 to 0.24 | .69 |
| Ventilator days | 0 [0-2] | 0 [0-2] | 0 [0-2] | 0 | 0-0 | .04 |
| TRU minutes | 451 [281-765] | 466 [290-798] | 432 [262-734] | 35 | 11-59 | <.001 |
| Total ICU Days | 0 [0-3.3] | 0 [0-2.5] | 0 [0-4.4] | 0 | 0-0 | .03 |
| Glasgow Coma Scale | 14 [11-15] | 14 [12-15] | 14 [10-15] | 0 | 0-0 | .16 |
| PTA Glasgow Coma Scale | 13 [7-15] | 13 [8-15] | 13 [7-15] | 0 | 0-0 | .27 |
| Injury Severity Score | 17 [10-25] | 17 [10-25] | 17 [10-25] | 0 | 0-0 | .37 |
| TRISS | 0.95 [0.88-0.98] | 0.95 [0.88-0.98] | 0.95 [0.88-0.98] | 0.002 | 0-0.005 | .03 |
| White Blood Cell Count (×1000 counts/μL) | 9.9 [7.5-13.4] | 9.8 [7.4-13.2] | 10.0 [7.6-13.6] | -0.2 | -0.5 to 0.1 | .29 |
| Admission SBP | 145 [130-169] | 145 [130-169] | 144 [129-170] | 0 | -2 to 2 | .74 |
| Admission DBP | 84 [74-94] | 84 [74-94] | 84 [74-94] | 0 | -1 to 1 | .91 |
| Procedures | | | | | | |
| Craniotomy | 118 (4) | 55 (3) | 63 (5) | -0.02 | -0.03 to -0.003 | .02 |
| Craniectomy | 17 (0.6) | 7 (0.4) | 10 (1) | -0.004 | -0.01 to 0.002 | .19 |
| Burr hole | 6 (0.2) | 1 (0) | 5 (0.4) | -0.004 | -0.007 to 0.0003 | .09 |
| Cranioplasty | 12 (0.4) | 4 (0.2) | 8 (0.7) | -0.004 | -0.009 to 0.001 | .11 |
| External Ventricular Drain | 65 (2) | 34 (2) | 31 (3) | -0.005 | -0.02 to 0.006 | .39 |
| CT Scan of Head | 2,546 (90) | 1,466 (91) | 1,080 (90) | 0.001 | -0.02 to 0.02 | .89 |
| X-Ray of Head | 13 (0.5) | 9 (0.5) | 4 (0.3) | 0.002 | -0.003 to 0.007 | .37 |
| Disposition | | | | | | |
| Death / Hospice | 319 (11) | 193 (12) | 126 (11) | 0.01 | -0.01, 0.04 | .25 |
| Non-home | 1,420 (50) | 824 (52) | 596 (50) | 0.01 | -0.03, 0.05 | .57 |

*MUA*, Medically Underserved Area; HPSA, Health Professional Shortage Area; *Diff*, unadjusted absolute difference between the two comparison groups; *CI*, confidence interval; *TRU*, trauma resuscitation unit; *ICU*, intensive care unit; *PTA GCS*, pre-transfer or pre-hospital Glasgow Coma Scale; *TRISS*, Trauma and Injury Severity Score; *SBP*, systolic blood pressure; *DBP*, diastolic blood pressure; *CT*, computed tomography

**Appendix E1**. Health professional shortage area baseline characteristics in a study of traumatic brain injury outcomes at a regional Level I trauma center.

| Variables | All  Patients  (N = 2,812) | HPSA | | | | |
| --- | --- | --- | --- | --- | --- | --- |
|  |  | Non-HPSA  (n = 1,377) | HPSA  (n = 1,435) | Diff | 95% CI | *P* |
| Demographics | | | | | | |
| Female (%) | 900 (32) | 485 (35) | 415 (29) | 0.06 | 0.03-0.10 | <.001 |
| Male (%) | 1,911 (68) | 892 (65) | 1019 (71) | -0.06 | -0.10 to -0.03 | <.001 |
| Age, median [IQR] | 60.5 [40-77] | 65 [44-80] | 57 [38-74] | 5 | 4-7 | <.001 |
| Median household income [$] | 89,398 [61,572-112,065] | 102,592 [82,242-122,021] | 68,626 [51,094-95,470] | 32,067 | 30,567-33,931 | <.001 |
| Rural (%) | 267 (9) | 62 (4) | 205 (14) | -0.10 | -0.12 to -0.08 | <.001 |
| Race / Ethnicity (%) | | | | | | |
| White | 1,731 (62) | 952 (69) | 779 (54) | 0.15 | 0.11-0.18 | <.001 |
| Black | 842 (30) | 306 (22) | 536 (37) | -0.15 | -0.18 to -0.12 | <.001 |
| Asian | 59 (2) | 37 (3) | 22 (2) | 0.01 | 0.001-0.02 | .03 |
| Native Hawaiian / Pacific Isl. | 4 (0.1) | 4 (0.3) | 0 (0) | 0.003 | 0.0-0.006 | .06 |
| American Indian / Alaskan Native | 6 (0.2) | 2 (0.1) | 4 (0.3) | -0.001 | -0.005 to 0.002 | .68 |
| Other / 2+ Races | 133 (5) | 62 (5) | 71 (5) | -0.004 | -0.02 to 0.01 | .58 |
| Comorbidities (%) | | | | | | |
| Hypertension | 1,298 (46) | 634 (46) | 664 (46) | -0.002 | -0.04 to 0.03 | .90 |
| Diabetes | 469 (17) | 220 (16) | 249 (17) | -0.01 | -0.04 to 0.01 | .32 |
| Liver disease | 125 (4) | 56 (4) | 69 (5) | -0.007 | -0.02 to 0.008 | .34 |
| Cardiac disease | 504 (18) | 238 (17) | 266 (19) | -0.01 | -0.04 to 0.02 | .39 |
| Kidney disease | 97 (3) | 47 (3) | 50 (3) | -0.001 | -0.01 to 0.01 | .92 |
| Neurological disorder | 744 (26) | 368 (27) | 376 (26) | 0.005 | -0.03 to 0.04 | .75 |
| Alcohol / drug use | 246 (9) | 130 (9) | 116 (8) | 0.01 | -0.007 to 0.03 | .20 |
| Prior surgery | 1,017 (36) | 479 (35) | 538 (37) | -0.03 | -0.06 to 0.008 | .13 |
| Top 5 Injury Mechanisms (%) | | | | | | |
| Fall | 1,777 (63) | 878 (64) | 899 (63) | 0.01 | -0.02 to 0.05 | .54 |
| ATV accident | 398 (14) | 205 (15) | 193 (13) | 0.01 | -0.01 to 0.04 | .27 |
| Assault | 329 (12) | 144 (10) | 185 (13) | -0.02 | -0.05 to -0.001 | .04 |
| Pedestrian accident | 101 (4) | 49 (4) | 52 (4) | -0.001 | -0.01 to 0.01 | .93 |
| Inanimate mechanical force | 63 (2) | 26 (2) | 37 (3) | -0.007 | -0.02 to 0.004 | .21 |

*HPSA*, Health Professional Shortage Area; *Diff*, unadjusted absolute difference between the two comparison groups; *CI*, confidence interval; *IQR*, interquartile range; *ATV*, all-terrain vehicle

**Appendix E2**. Health professional shortage area clinical course and outcomes in a study of traumatic brain injury outcomes at a regional Level I trauma center.

|  | All  Patients  (N = 2,812) | HPSA | | | | |
| --- | --- | --- | --- | --- | --- | --- |
|  |  | Non-HPSA  (n = 1,377) | HPSA  (n = 1,435) | Diff | 95% CI | *P* |
| Injury (%) | | | | | | |
| Subdural hematoma | 1,885 (67) | 922 (67) | 963 (67) | -0.002 | -0.04 to 0.03 | .93 |
| Subarachnoid Hemorrhage | 1,251 (44) | 591 (43) | 660 (46) | -0.03 | -0.07 to 0.005 | .10 |
| Epidural Hematoma | 289 (10) | 135 (10) | 154 (11) | -0.009 | -0.03 to 0.01 | .42 |
| Edema | 239 (8) | 116 (8) | 123 (8) | -0.001 | -0.02 to 0.02 | .89 |
| Bacteremia | 4 (0.1) | 1 (0) | 3 (0.2) | -0.001 | -0.004 to 0.001 | .63 |
| Clinical Variables | | | | | | |
| Length of Stay (days) | 3.6 [1.1-8.8] | 3.6 [1.1-9.0] | 3.3 [0.84-7.8] | 0.21 | -0.10 to 0.61 | .19 |
| Ventilator days | 0 [0-2] | 0 [0-2] | 0 [0-2] | 0 | 0-0 | .93 |
| TRU minutes | 451 [281-765] | 451 [283-771] | 444 [255-700] | 23.5 | -16.7 to 64.4 | .25 |
| Total ICU Days | 0 [0-3.3] | 0 [0-3.3] | 0 [0-2.7] | 0 | 0-0 | .58 |
| Glasgow Coma Scale | 14 [11-15] | 14 [11-15] | 14 [9-15] | 0 | 0-0 | .54 |
| PTA Glasgow Coma Scale | 13 [7-15] | 13 [7-15] | 13 [7-14] | 0 | 0-1 | .17 |
| Injury Severity Score | 17 [10-25] | 17 [10-25] | 16 [10-25] | 0 | 0-0 | .50 |
| TRISS | 0.95 [0.88-0.98] | 0.95  [0.88-0.98] | 0.95 [0.87-0.98] | 0 | -0.002 to 0.005 | .68 |
| White Blood Cell Count (×1000 counts/μL) | 9.9 [7.5-13.4] | 9.9 [7.4-13.4] | 10.4 [7.8-13.2] | -0.2 | -0.7 to 0.3 | .47 |
| Admission SBP | 145 [130-169] | 145 [130-170] | 144 [129-166] | 2 | -2 to 5 | .30 |
| Admission DBP | 84 [74-94] | 84 [73-94] | 84 [73-94] | 0 | -2 to 3 | .65 |
| Procedures | | | | | | |
| Craniotomy | 118 (4) | 55 (4) | 63 (4) | -0.004 | -0.02 to 0.01 | .60 |
| Craniectomy | 17 (0.6) | 7 (0.5) | 10 (0.7) | -0.002 | -0.007 to 0.004 | .52 |
| Burr hole | 6 (0.2) | 2 (0.1) | 4 (0.3) | -0.001 | -0.005 to 0.002 | .69 |
| Cranioplasty | 12 (0.4) | 5 (0.4) | 7 (0.5) | -0.001 | -0.006 to 0.004 | .61 |
| External Ventricular Drain | 65 (2) | 30 (2) | 35 (2) | -0.003 | -0.01 to 0.008 | .65 |
| CT Scan of Head | 2,546 (90) | 1,250 (91) | 1,296 (90) | -0.005 | -0.02 to 0.03 | .67 |
| X-Ray of Head | 13 (0.5) | 7 (0.5) | 6 (0.4) | 0.001 | -0.004 to 0.006 | .72 |
| Disposition | | | | | | |
| Death/Hospice | 319 (11) | 154 (11) | 165 (11) | -0.003 | -0.03 to 0.02 | .79 |
| Non-home | 1,420 (50) | 694 (51) | 726 (51) | -0.004 | -0.04 to 0.03 | .84 |

*HPSA*, Health Professional Shortage Area; *Diff*, unadjusted absolute difference between the two comparison groups; *CI*, confidence interval; *TRU*, trauma resuscitation unit; *ICU*, intensive care unit; *PTA GCS*, pre-transfer or pre-hospital Glasgow Coma Scale; *TRISS*, Trauma and Injury Severity Score; *SBP*, systolic blood pressure; *DBP*, diastolic blood pressure; *CT*, computed tomography

**Appendix F1**. Maryland health enterprise zone baseline characteristics in a study of traumatic brain injury outcomes at a regional Level I trauma center.

| Variables | All  Patients  (N = 2,812) | MHEZ | | | | |
| --- | --- | --- | --- | --- | --- | --- |
|  |  | Non-MHEZ  (n = 2,467) | MHEZ  (n = 345) | Diff | 95% CI | *P* |
| Demographics | | | | | | |
| Female (%) | 900 (32) | 804 (33) | 96 (28) | 0.05 | -0.004 to 0.10 | .07 |
| Male (%) | 1,911 (68) | 1,663 (67) | 248 (72) | -0.05 | -0.10 to 0.004 | .07 |
| Age, median [IQR] | 60.5 [40-77] | 61 [39-78] | 58 [44-71] | 2 | -1 to 4 | .13 |
| Median household income [$] | 89,398 [61,572-112,065] | 94,815 [77,398-115,807] | 46,761 [36,665-52,692] | 43,769 | 42,716-45,312 | <.001 |
| Rural (%) | 267 (9) | 225 (9) | 42 (12) | -0.03 | -0.07 to 0.01 | .10 |
| Race / Ethnicity (%) | | | | | | |
| White | 1,731 (62) | 1,615 (65) | 116 (34) | 0.32 | 0.27-0.37 | <.001 |
| Black | 842 (30) | 630 (25) | 212 (61) | -0.36 | -0.41 to -0.30 | <.001 |
| Asian | 59 (2) | 57 (2) | 2 (0.6) | 0.02 | 0.007-0.03 | .04 |
| Native Hawaiian / Pacific Isl. | 4 (0.1) | 4 (0.2) | 0 (0) | 0.002 | 0.0-0.003 | >.99 |
| American Indian / Alaskan Native | 6 (0.2) | 5 (0.2) | 1 (0.2) | -0.001 | -0.007 to 0.005 | .54 |
| Other / 2+ Races | 133 (5) | 123 (5) | 10 (3) | 0.02 | 0.001-0.04 | .04 |
| Comorbidities (%) | | | | | | |
| Hypertension | 1,298 (46) | 1,124 (45) | 174 (50) | -0.05 | -0.10 to 0.007 | .09 |
| Diabetes | 469 (17) | 412 (17) | 57 (17) | 0.002 | -0.04 to 0.04 | .93 |
| Liver disease | 125 (4) | 113 (4) | 12 (3) | 0.01 | -0.01 to 0.03 | .30 |
| Cardiac disease | 504 (18) | 439 (18) | 65 (19) | -0.01 | -0.05 to 0.03 | .64 |
| Kidney disease | 97 (3) | 85 (3) | 12 (3) | -0.0003 | -0.02 to 0.02 | .98 |
| Neurological disorder | 744 (26) | 639 (26) | 105 (30) | -0.04 | -0.10 to 0.006 | .08 |
| Alcohol / drug use | 246 (9) | 221 (9) | 25 (7) | 0.02 | -0.01 to 0.05 | .26 |
| Prior surgery | 1,017 (36) | 881 (36) | 136 (39) | -0.04 | -0.09 to 0.02 | .19 |
| Top 5 Injury Mechanisms (%) | | | | | | |
| Fall | 1,777 (63) | 1,554 (63) | 223 (65) | -0.02 | -0.07 to 0.04 | .55 |
| ATV accident | 398 (14) | 352 (14) | 46 (13) | 0.009 | -0.03 to 0.05 | .63 |
| Assault | 329 (12) | 292 (12) | 37 (11) | 0.01 | -0.02 to 0.05 | .53 |
| Pedestrian accident | 101 (4) | 89 (4) | 12 (3) | 0.001 | -0.02 to 0.02 | .90 |
| Inanimate mechanical force | 63 (2) | 56 (2) | 7 (2) | 0.002 | -0.01 to 0.02 | .77 |

MHEZ, Maryland Health Enterprise Zone; Diff, unadjusted absolute difference between the two comparison groups; CI, confidence interval; IQR, interquartile range; ATV, all-terrain vehicle

**Appendix F2**. Maryland health enterprise zone clinical course and outcomes in a study of traumatic brain injury outcomes at a regional Level I trauma center.

|  | All  Patients  (N = 2,812) | MHEZ | | | | |
| --- | --- | --- | --- | --- | --- | --- |
|  |  | Non-MHEZ  (n = 2,467) | MHEZ  (n = 345) | Diff | 95% CI | *P* |
| Injury (%) | | | | | | |
| Subdural hematoma | 1,885 (67) | 1,639 (66) | 246 (71) | -0.05 | -0.10 to 0.003 | .06 |
| Subarachnoid Hemorrhage | 1,251 (44) | 1,081 (44) | 170 (49) | -0.05 | -0.11 to 0.002 | <.001 |
| Epidural Hematoma | 289 (10) | 246 (10) | 43 (12) | -0.02 | -0.06 to 0.01 | .18 |
| Edema | 239 (8) | 209 (8) | 30 (9) | -0.002 | -0.03 to 0.03 | .89 |
| Bacteremia | 88 (3) | 77 (3) | 11 (3) | -0.001 | -0.02 to 0.02 | .95 |
| Clinical Variables | | | | | | |
| Length of Stay (days) | 3.6 [1.1-8.8] | 3.5 [1.1-8.6] | 4.1 [0.96-10.2] | -0.09 | -0.44 to 0.21 | .54 |
| Ventilator days | 0 [0-2] | 0 [0-2] | 0 [0-2] | 0 | 0-0 | .37 |
| TRU minutes | 451 [281-765] | 453 [286-764] | 439 [246-774] | 25 | -11 to 60.5 | .18 |
| Total ICU Days | 0 [0-3.3] | 0 [0-3.1] | 0 [0-4.0] | 0 | 0-0 | .45 |
| Glasgow Coma Scale | 14 [11-15] | 14 [11-15] | 14 [11-15] | 0 | 0-0 | .90 |
| PTA Glasgow Coma Scale | 13 [7-15] | 13 [8-15] | 14 [7-15] | 0 | 0-0 | .50 |
| Injury Severity Score | 17 [10-25] | 17 [10-25] | 17 [10-25] | 0 | 0-0 | .49 |
| TRISS | 0.95 [0.88-0.98] | 0.95 [0.88-0.98] | 0.94 [0.88-0.97] | 0.001 | 0-0.008 | .21 |
| White Blood Cell Count (×1000 counts/μL) | 9.9 [7.5-13.4] | 9.8 [7.4-13.2] | 10.5 [8.0-14.2] | -0.6 | -1.1 to -0.1 | .02 |
| Admission SBP | 145 [130-169] | 145 [130-169] | 143 [129-169] | 2 | -1 to 5 | .29 |
| Admission DBP | 84 [74-94] | 84 [74-94] | 82 [73-92] | 1 | -1 to 3 | .20 |
| Procedures | | | | | | |
| Craniotomy | 118 (4) | 107 (4) | 11 (3) | 0.01 | -0.009 to 0.03 | .26 |
| Craniectomy | 17 (0.6) | 17 (0.6) | 0 (0) | 0.007 | 0.004-0.01 | .25 |
| Burr hole | 6 (0.2) | 5 (0.2) | 1 (0.3) | -0.001 | -0.007 to 0.005 | .54 |
| Cranioplasty | 12 (0.4) | 9 (0.4) | 3 (0.9) | -0.005 | -0.02 to 0.005 | .17 |
| External Ventricular Drain | 65 (2) | 55 (2) | 10 (3) | -0.007 | -0.03 to 0.01 | .48 |
| CT Scan of Head | 2,546 (90) | 2,224 (90) | 322 (93) | -0.03 | -0.06 to -0.003 | .03 |
| X-Ray of Head | 13 (0.5) | 11 (0.4) | 2 (0.5) | -0.001 | -0.01 to 0.007 | .67 |
| Disposition | | | | | | |
| Death / Hospice | 319 (11) | 282 (11) | 37 (11) | 0.007 | -0.03 to 0.04 | .69 |
| Non-home | 1,420 (50) | 1,236 (51) | 184 (54) | -0.03 | -0.09 to 0.03 | .30 |

MHEZ, Maryland Health Enterprise Zone; Diff, unadjusted absolute difference between the two comparison groups; CI, confidence interval; *TRU*, trauma resuscitation unit; *ICU*, intensive care unit; *PTA GCS*, pre-transfer or pre-hospital Glasgow Coma Scale; *TRISS*, Trauma and Injury Severity Score; *SBP*, systolic blood pressure; *DBP*, diastolic blood pressure; *CT*, computed tomography

**Appendix G**. State Police Aviation Command helicopter sections (Trooper 1-7) and base locations in a study of traumatic brain injury outcomes at a regional Level I trauma center.

Source: *Maryland Police Aviation Command, Adapted from* https://mdsp.maryland.gov/Organization/Pages/SupportServicesBureau/AviationCommand/Sections.aspx *Red Star marks the location of the STC.*
